# Ultrasound parameters associated with stroke in patients with moyamoya disease: a logistic regression analysis

**DOI:** 10.1186/s41016-022-00300-5

**Published:** 2022-10-11

**Authors:** Shuai Zheng, Fumin Wang, Linggang Cheng, Rui Li, Dong Zhang, Wen He, Wei Zhang

**Affiliations:** 1 Department of Ultrasound, Beijing Tiantan Hospital, Capital Medical University, No. 119, West Road of South Fourth Ring, Fengtai District, Beijing, 100070 People’s Republic of China; 2 Department of Ultrasound, Peking University First Hospital, No. 8, Xishiku street, Xicheng District, Beijing, 100034 People’s Republic of China; 3 Department of Neurosurgery, Beijing Hospital, No.1, Dahua Road, Dongdan, Dongcheng District, Beijing, 100730 People’s Republic of China; 4 Department of Neurosurgery, Beijing Tiantan Hospital, Capital Medical University, No. 119, West Road of South Fourth Ring, Fengtai District, Beijing, 100070 People’s Republic of China

**Keywords:** Ultrasound, Moyamoya disease, Stroke, Internal carotid artery, Posterior cerebral artery

## Abstract

**Background:**

Moyamoya disease can lead to stroke with devastating consequences, it is necessary to find a non-invasive and effective approach to identify the occurrence of stroke. In this study, we aim to analyze the association between ultrasound parameters and ipsilateral cerebral hemisphere stroke in patients with moyamoya disease by logistic regression analysis.

**Methods:**

In this retrospective case–control study, 88 patients with MMD (153 cerebral hemispheres) hospitalized in Beijing Tiantan Hospital, Capital Medical University from November 2020 to October 2021 were analyzed. According to the occurrence of stroke, the 153 cerebral hemispheres were divided into a stroke group and a non-stroke group. Clinical data and ultrasound parameters of the ipsilateral internal carotid artery, superficial temporal artery, maxillary artery, and posterior cerebral artery were recorded. The ultrasound parameters were divided into four groups according to interquartile range, and then they were compared between the stroke group and the non-stroke group. Those with significant differences were scored by multivariate logistic regression analysis.

**Results:**

There were 75 cerebral hemispheres (49.0%) in the stroke group and 78 cerebral hemispheres (51.0%) in the non-stroke group. Logistic regression analysis showed that the internal diameter of the internal carotid artery, peak systolic velocity of the internal carotid artery and peak systolic velocity of the posterior cerebral artery were independently correlated factors for stroke in patients with MMD. The fourth quartile group of the above three ultrasound parameters was taken as the reference group, and the odds ratio of the first quartile group were 11.679 (95% CI 2.918–46.749, *P* = 0.001), 19.594 (95% CI 4.973–77.193, *P* < 0.001), and 11.657 (95% CI 3.221–42.186, *P* < 0.001), respectively.

**Conclusion:**

Ultrasound parameters are independently correlated with ipsilateral cerebral stroke in patients with MMD. Ultrasound provides a new way to identify stroke in MMD patients. Future prospective cohort studies are needed to verify the clinical value of ultrasound in identifying patients with MMD at high risk of stroke.

## Background

Moyamoya disease (MMD) is a disease with unknown etiology, characterized by progressive stenosis or occlusion of terminals of the bilateral internal carotid artery (ICA) and its proximal branches, accompanied by the formation of abnormal vasoganglions [1]. The main clinical symptoms of MMD include ischemic stroke induced by cerebral ischemia, and intracerebral hemorrhage caused by the rupture of fragile collateral vessels [2] that response to cerebral ischemia. MMD is an important etiology of stroke in youth, middle-aged people, and children. Studies have pointed out that routine imaging monitoring of MMD patients can lower the burden of stroke and improve their clinical outcome [2, 3].

Digital subtraction angiography (DSA) is the gold standard for the diagnosis of MMD. According to the degree of luminal stenosis and the scope of involved intracranial vessels, as well as the density of moyamoya vessels in MMD patients, Suzuki et al. [1] proposed Suzuki stage, which has been widely applied in clinical practice. However, due to the existence of collateral circulation, some MMD patients with the same Suzuki stage may show different clinical symptoms [1, 2]. The leptomeningeal collateral (LMC) derived from the posterior cerebral artery (PCA) comprises the main collateral vessels of MMD, while the dural-leptomeningeal collaterals derived from the maxillary artery (MA) and superficial temporal artery (STA) can also supply blood for ischemic brain tissues of MMD patients [1, 4, 5]. DSA examination deepens our understanding of moyamoya disease, but it is limited in terms of the monitoring and follow-up of MMD because of its invasiveness, high cost and radiation exposure. As ultrasound enables us to acquire hemodynamic information from MMD patients in a non-invasive and convenient manner, it has been used in preoperative examination and postoperative prognosis evaluation of MMD [6–8]. However, systematic and in-depth studies on the relationship between ultrasound parameters and stroke in MMD patients is still lacking. In this work, we used logistic regression to analyze the relationship between ultrasound parameters including the ICA, STA, MA and PCA and ipsilateral cerebral hemisphere stroke in MMD patients, which provides evidence for the use of ultrasound to aid in identifying stroke in MMD patients.

## Methods

### Study population

In this retrospective case–control study, patients diagnosed with MMD by DSA in the Department of Neurosurgery of Beijing Tiantan Hospital from November 2020 to October 2021 were selected for ultrasound examination. The sonographer was blinded to the clinical data before examination. The present study was approved by the Ethics Committee. All adult patients and guardians of pediatric patients signed informed consent forms.

**Fig. 1:**
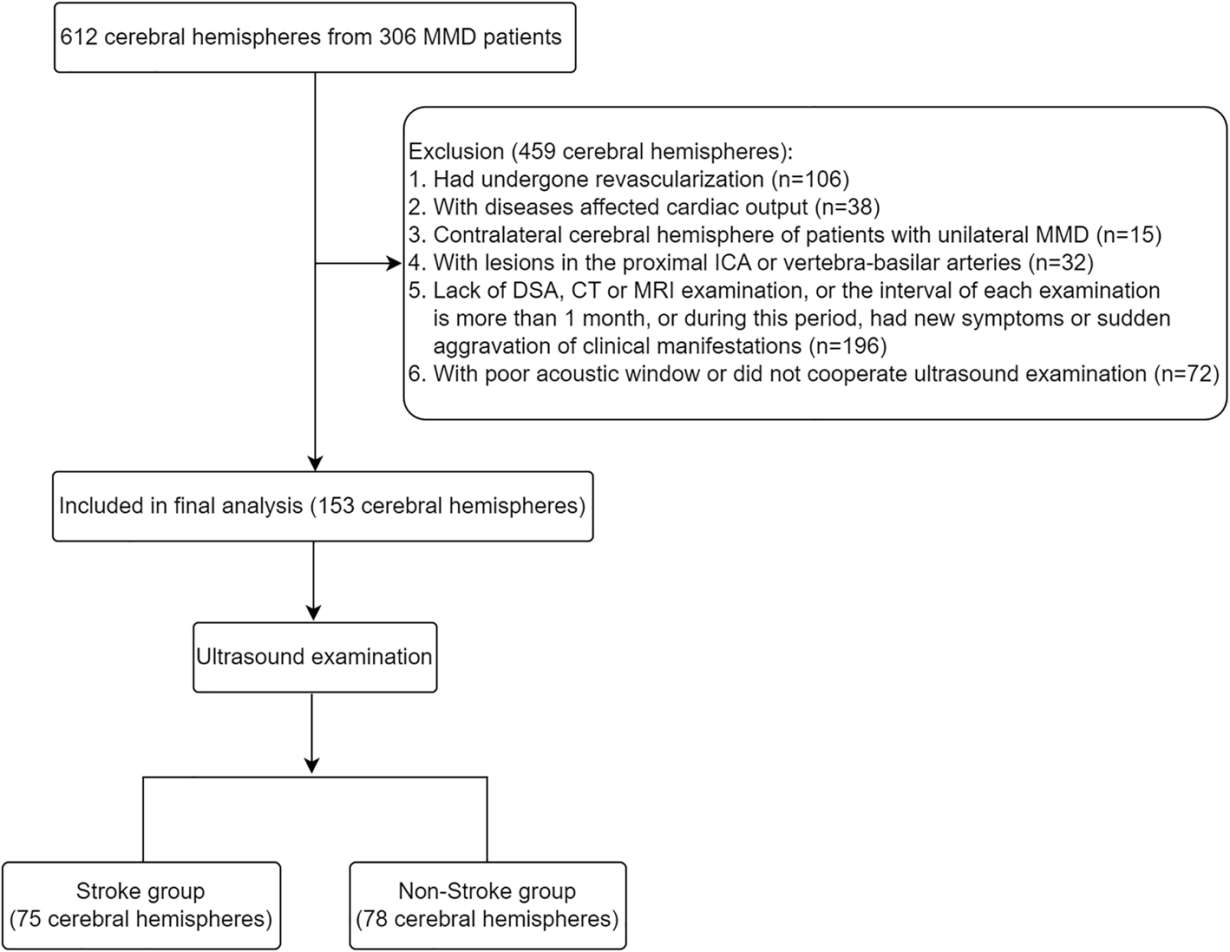
Flowchat of the study. *MMD* moyamoya disease, *ICA* internal carotid artery, *DSA* digital subtraction angiography, *CT* computed tomography*, MRI* magnetic resonance imaging

Inclusion criteria were as follows: (1) Patients who were diagnosed with MMD according to the diagnostic guidelines for MMD [9]; (2) MMD patients who underwent computed tomography (CT), MRI, DSA, and ultrasound examinations with an interval of less than 1 month between examinations. During this period, patients showed no new symptoms or sudden aggravation of clinical manifestations. Exclusion criteria included (1) MMD patients who underwent cerebral revascularization in the ipsilateral cerebral hemisphere; (2) MMD patients with diseases that affect cardiac output, such as severe anemia, hyperthyroidism, and congestive heart failure; (3) MMD patients with lesions in the proximal ICA or vertebra-basilar arteries; (4) MMD patients with poor sonolucency in the temporal window or those who did not cooperate in the ultrasound examination (Fig. 1).

### Diagnostic criteria for stroke and non-stroke

1. Stroke includes ischemic stroke and hemorrhagic stroke. Ischemic stroke refers to a new symptom of neurological deterioration, which is triggered by cerebral infarction and confirmed by MRI, while hemorrhagic stroke refers to intracranial hemorrhage confirmed by CT [10];
2. Non-stroke patients were those who did not have cerebral infarction or intracerebral hemorrhage. All clinical manifestations were confirmed by a neurosurgeon with more than 5 years of experience.

### Ultrasound examination

All ultrasound images were collected using the same ultrasound machine (Aplio 900, Canon Medical Systems Corporation, Japan). All subjects were examined by an experienced sonographer before surgical revascularization. Thirty patients were randomly selected to assess inter-operator consistency.

### ICA ultrasound examination

A 5–14 MHz linear array probe was used for ICA ultrasound examination. The patient took a supine position, with their head tilted back and slightly leaned toward the opposite side. On the longitudinal image of the carotid artery, the internal diameter (ID) of the ICA was measured 1–2 cm above the bulb of the carotid artery. Then, color Doppler and pulse wave Doppler were used to measure the peak systolic velocity (PSV) of the ICA.

### STA ultrasound examination

A 5–14 MHz linear array probe was used for STA ultrasound examination. The patient was placed in a supine position, and their head was turned to the other side. The probe was placed in front of their ear. The ID of the STA was measured in the longitudinal image of the STA. Then, color Doppler and pulse wave Doppler were used to measure the PSV of the STA.

### MA ultrasound examination

A 1–8 MHz convex array probe was used for MA ultrasound examination. The patient took a supine position and turned their head to the other side. The probe was placed at the mandibular angle and pointed at the tip of the nose. The PSV of the MA was measured using color Doppler and pulse wave Doppler.

### PCA ultrasound examination

A 1–6 MHz phased array probe was used for PCA ultrasound examination. The patient was placed in a lateral position, and the P2 segment of the PCA was examined through the temporal window. The PSV of the PCA was measured using color Doppler and pulse wave Doppler.

### Statistical analysis

Continuous variables that followed a normal distribution are expressed as the mean ± standard deviation (SD), and those did not follow a normal distribution are expressed as the median (interquartile range). We used an independent t test for continuous data following a normal distribution and Mann‒Whitney U test for continuous data not following a normal distribution. Count data are expressed as percentages, and the χ2 test was used for comparisons between groups. Parameters related to ipsilateral stroke in MMD patients were screened by multivariate logistic regression analysis. The interclass correlation coefficient (ICC) was adopted to analyze inter-examiner consistency. All hypothesis tests were two-tailed, with a *P* value < 0.05 indicating statistical significance. The statistical analysis software SPSS 25.0 was used.

## Results

### Comparison of baseline data between the stroke group and non-stroke group

A total of 153 cerebral hemispheres from 88 MMD patients were included in this analysis, including 75 cerebral hemispheres (49.0%) in the stroke group and 78 cerebral hemispheres (51.0%) in the non-stroke group. There were no significant differences between the two groups in terms of sex, age, or the proportions of patients with hypertension, diabetes, hyperlipidemia, smoking and drinking (Table 1).

**Table 1:** Comparison of baseline data between the stroke group and non-stroke group for MMD

Characteristics	Stroke group (*n* = 75 hemispheres)	Non-Stroke group (*n* = 78 hemispheres)	*P* value
Sex, male, (%)	32(42.7)	33(42.3)	0.964
Age, years	44 (33–52)	41 (32–48)	0.083
Clinical history, (%)			
Hypertension	23(30.6)	26(33.3)	0.724
Diabetes	7(9.3)	7(9.0)	0.939
Hyperlipidemia	9(12.0)	5(6.4)	0.231
Smoking	12(16.0)	8(10.3)	0.292
Drinking	9(12.0)	9(11.5)	0.929

### Inter-operator consistency test

ICC was used to compare the ID of the ICA, the PSV of the ICA, the ID of the STA, the PSV of the STA, the PSV of the MA and the PSV of the PCA measured by 2 different operators, and the consistency was good for the above parameters (ICCs were 0.875, 0.935, 0.892, 0.900, 0.898 and 0.913, respectively).

### Comparison of ultrasound parameters between the stroke group and non-stroke group

**Fig. 2:**
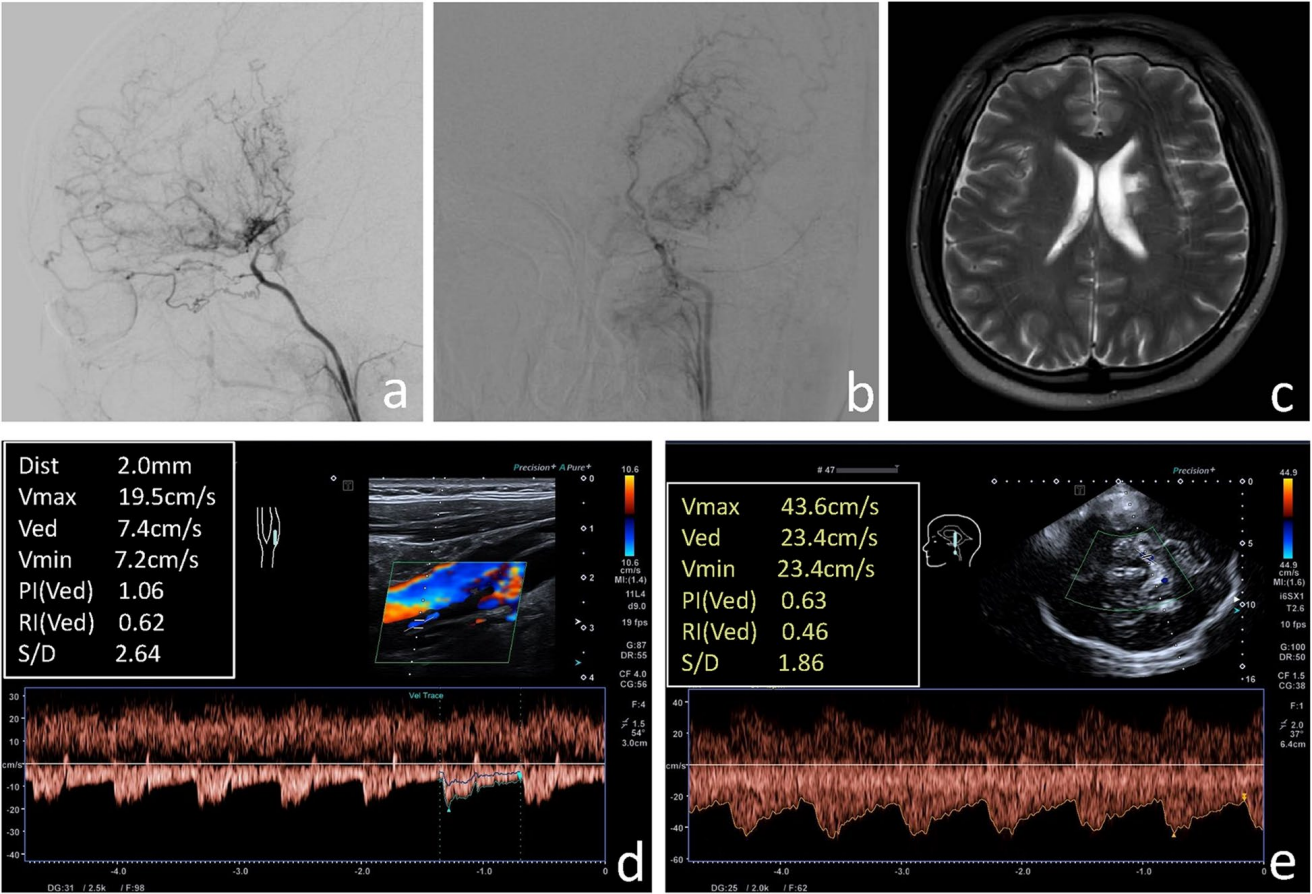
**a, b** Digital subtraction angiography confirmed moyamoya disease in the left hemisphere. **c** Brain magnetic resonance imaging showed an infarct in the left hemisphere. **d **Ultrasound examination of the left internal carotid artery. **e** Transcranial color-coded duplex sonography of the left posterior cerebral artery

**Fig. 3:**
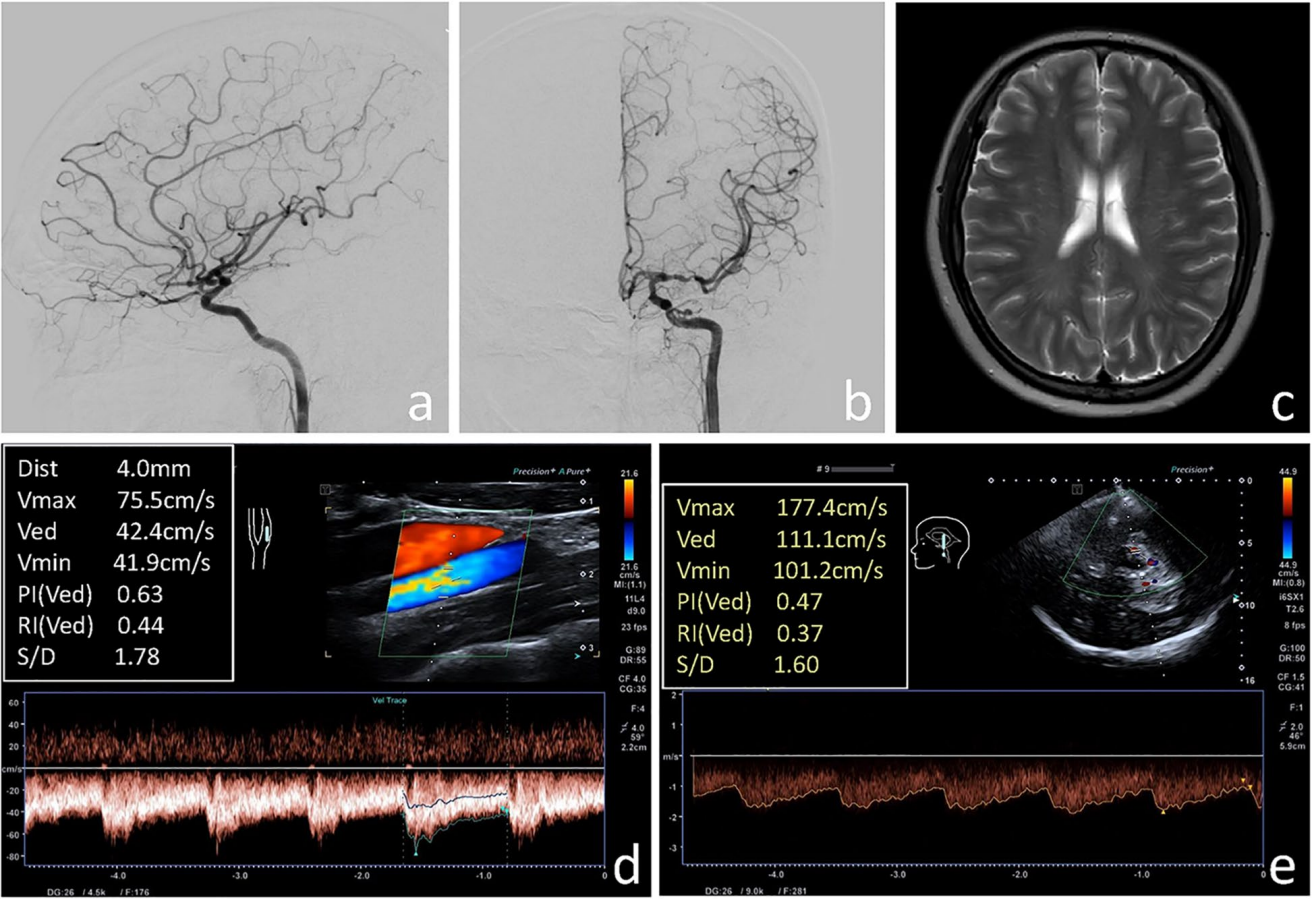
**a, b** Digital subtraction angiography confirmed moyamoya disease in the left hemisphere. **c** Brain magnetic resonance imaging showed no obvious abnormalities in the left hemisphere. **d** Ultrasound examination of the left internal carotid artery. **e** Transcranial color-coded duplex sonography of the left posterior cerebral artery

The ultrasound parameters, including the ID of the ICA, the PSV of the ICA, the ID of the STA, the PSV of the STA, the PSV of the MA and the PSV of the PCA were divided into four groups according to interquartile range, and the differences between the stroke group and the non-stroke group in various ultrasound parameters were compared. The differences between the stroke group and the non-stroke group in the ID of the ICA, the PSV of the ICA and the PSV of the PCA were statistically significant (all *P* < 0.001), the differences in other ultrasound parameters between the two groups were not significant (Table 2) (Figs. 2 and 3).

**Table 2:** Comparison of ultrasound parameters between the stroke group and non-stroke group for MMD

Variables	Stroke group (*n* = 75 hemispheres)	Non-Stroke group (*n* = 78 hemispheres)	*P* value
ICA-ID (cm)			< 0.001
Q1(< 0.30)	25 (33.3)	6 (7.7)	
Q2(0.30–0.33)	18 (24.0)	18 (23.1)	
Q3(0.34–0.37)	21 (28.0)	23 (29.5)	
Q4(≥ 0.38)	11 (14.7)	31 (39.7)	
ICA-PSV (cm/s)			< 0.001
Q1(< 41.85)	29 (38.6)	9 (11.5)	
Q2(41.85–57.59)	21 (28.0)	17 (21.8)	
Q3(57.60–75.09)	17 (22.7)	22 (28.2)	
Q4(≥ 75.10)	8 (10.7)	30 (38.5)	
STA-ID (cm)			0.774
Q1(< 0.16)	26 (34.6)	26 (33.4)	
Q2(0.16–0.17)	27 (36.0)	27 (34.6)	
Q3(0.18–0.18)	5 (6.7)	9 (11.5)	
Q4(≥ 0.19)	17 (22.7)	16 (20.5)	
STA-PSV (cm/s)			0.557
Q1(< 59.80)	15 (20.0)	23 (29.4)	
Q2(59.80–73.99)	19 (25.3)	19 (24.4)	
Q3(74.00–87.09)	20 (26.7)	19 (24.4)	
Q4(≥ 87.10)	21 (28.0)	17 (21.8)	
MA-PSV (cm/s)			0.556
Q1(< 45.60)	22 (29.3)	16 (20.5)	
Q2(45.60–51.59)	16 (21.3)	22 (28.1)	
Q3(51.60–63.99)	17 (22.7)	20 (25.6)	
Q4(≥ 64.00)	20 (26.7)	20 (25.6)	
PCA-PSV (cm/s)			< 0.001
Q1(< 84.60)	30 (40)	8 (10.2)	
Q2(84.60–98.19)	20 (26.6)	18 (23.1)	
Q3(98.20–137.49)	17 (22.7)	22 (28.2)	
Q4(≥ 137.50)	8 (10.7)	30 (38.5)	

*ICA-ID* Internal diameter of internal carotid artery, *Q1* First quartile, *Q2* Second quartile, *Q3* Third quartile, *Q4* Fourth quartile, *ICA-PSV* Peak systolic velocity of internal carotid artery, *STA-ID* Internal diameter of superficial temporal artery, *STA-PSV* Peak systolic velocity of superficial temporal artery, *MA-PSV* Peak systolic velocity of maxillary artery, *PCA-PSV* Peak systolic velocity of posterior cerebral artery

### Multivariate logistic regression analysis

Logistic regression analysis showed that the ID of the ICA, the PSV of the ICA and the PSV of the PCA were independent factors related to ipsilateral cerebral hemisphere stroke in MMD. Taking the fourth percentile group of the ID of ICA as the reference group, the OR values of the first and second percentile groups of ICA were 11.679 (95%CI 2.918–46.749, *P* = 0.001) and 4.660 (95%CI 1.480–14.673, *P* = 0.009), respectively. Taking the fourth percentile group of the PSV of ICA as the reference group, the OR values of the first, second and third percentile groups of the PSV of ICA were 19.594 (95%CI 4.973–77.193, *P* < 0.001), 7.988 (95%CI 2.194–29.086, *P* = 0.002) and 3.665 (95%CI 1.079–12.454, *P* = 0.037), respectively. Taking the fourth percentile group of the PSV of PCA as the reference group, the OR values of the first and second percentile groups of PSV of PCA were 11.657 (95% CI 3.221–42.186, *P* < 0.001) and 3.875 (95% CI 1.197–12.552, *P* = 0.024), respectively (Table 3).

**Table 3:** Multivariate logistic regression analysis of ultrasound parameters related to stroke for MMD

Variables	Multivariate logistic regression analysis		
	OR	(95% CI)	*P* value
ICA-ID (cm)			
Q4(≥ 0.38)	1	-	-
Q3(0.34–0.37)	2.178	0.737–6.433	0.159
Q2(0.30–0.33)	4.660	1.480–14.673	0.009
Q1(< 0.30)	11.679	2.918–46.749	0.001
ICA-PSV (cm/s)			
Q4(≥ 75.10)	1	-	-
Q3(57.60–75.09)	3.665	1.079–12.454	0.037
Q2(41.85–57.59)	7.988	2.194–29.086	0.002
Q1(< 41.85)	19.594	4.973–77.193	< 0.001
PCA-PSV (cm/s)			
Q4(≥ 137.50)	1	-	-
Q3(98.20–137.49)	2.072	0.655–6.557	0.215
Q2(84.60–98.19)	3.875	1.197–12.552	0.024
Q1(< 84.60)	11.657	3.221–42.186	< 0.001

*OR* Odds ratio, *CI* Confidence interval, *ICA-ID* Internal diameter of internal carotid artery, *Q4* Fourth quartile, *Q3* Third quartile, *Q2* Second quartile, *Q1* First quartile, *ICA-PSV* Peak systolic velocity of internal carotid artery, *PCA-PSV* Peak systolic velocity of posterior cerebral artery

## Discussion

In this study, we analyzed the relationship between ultrasound parameters and ipsilateral cerebral hemisphere stroke in MMD patients. The results showed that the ID of the ICA, the PSV of the ICA and the PSV of the PCA were independently related to the occurrence of ipsilateral cerebral hemisphere stroke in MMD patients. Ultrasound has provided a new method for identifying stroke in MMD patients. At present, DSA is the gold standard for the diagnosis of MMD. Japanese scholars Suzuki and Takaku [1] divided MMD into six stages according to the degree of the luminal stenosis and the scope of the involved intracranial arteries, as well as the density of moyamoya vessels in DSA images. Although DSA examination is helpful for long-term monitoring of MMD and assessing the risk of adverse clinical outcomes, its clinical application is limited due to its invasiveness, radioactivity, and the need to inject exogenous contrast agents. Ultrasound is a non-invasive, non-radiative and economical examination method and has been used in the screening of MMD, preoperative detection of compensatory vessels and postoperative prognosis evaluation of MMD [6–8].

Yasuda et al. [11] used carotid ultrasound to measure the internal diameter of the ICA and the common carotid artery, and the results showed that with the progression of MMD and the reduction in cerebrovascular reactivity, the ratio of the ICA diameter to the common carotid artery diameter decreased. When the ratio of the two was less than 0.5, it was called the “bottleneck sign”. Patients with the “bottleneck sign” were more prone to have ipsilateral cerebrovascular events. Wang et al. [12] reported that patients with ipsilateral intracranial hemorrhage had a lower ratio of the ICA diameter to the common carotid artery diameter than those patients without intracranial hemorrhage. The incidence of ipsilateral intracranial hemorrhage in patients with the “bottleneck sign” was significantly higher than that in those without the “bottleneck sign”. The “bottleneck sign” was significantly associated with ipsilateral intracranial hemorrhage in MMD patients. Our findings coincided with those of Yasuda [11] and Wang et al. [12]. We discovered that the ID of the ICA and the PSV of the ICA were independent parameters related to ipsilateral cerebral hemisphere stroke in MMD patients. With the progression of MMD, the ID of the ICA gradually decreases [11, 13], and the velocity of the ICA also decreases due to the increase in resistance in the distal ICA. The decrease in the ID and PSV of the ICA leads to hypoperfusion in the ACA and MCA regions [14]. Hypoperfusion increases susceptibility to ischemia, and abnormal hemodynamics may result in ischemic stroke [15]. Intracerebral hemorrhage is an adverse outcome of compensatory response to cerebral ischemia. Increased hemodynamic abnormalities lead to the rupture of fragile moyamoya vessels and Willis aneurysms, which may eventually lead to hemorrhagic stroke [16]. Therefore, with the progression of MMD, the ID and PSV of the ICA decreased, both of which are independent parameters related to ipsilateral cerebral hemisphere stroke in MMD patients.

Clinically, some MMD patients have the same Suzuki stage, but the severity of clinical symptoms is different. This may be attributed to the existence of collateral circulation, which supplies extra blood for ischemic brain tissues. The LMCs derived from the PCA are regarded as the main collateral vessels in MMD patients, while the dural-leptomeningeal collaterals derived from external carotid arteries can also supply blood for ischemic brain tissues [1, 5, 17, 18]. In this work, we used ultrasound to detect the hemodynamic parameters of the PCA, STA and MA, including the PSV of the PCA, the ID and PSV of the STA, and the PSV of the MA. Since the location of the PCA and the MA is relatively deep, phased array probe and convex array probe were used to detect PCA and MA respectively, which can't directly display the lumen structure. Therefore, we measured the velocity of the PCA and the MA to reflect the compensation for the anterior circulation.

We noticed that the PSV of the PCA was an independently related parameter for ipsilateral hemispheric stroke in MMD patients. In this work, we measured ultrasound parameters in the P2 segment of the PCA for study according to previous studies [19, 20]. We found that the higher the PSV of PCA, the less likely the patient suffered from ipsilateral cerebral hemisphere stroke. It was conjectured that the high PSV of the PCA can generate abundant collateral circulation. If the P1 segment of the PCA becomes narrow, the P2 segment would show low velocity and could not form abundant collateral circulation. Our result is consistent with those of previous studies. Previous studies reported that the wider the range of blood supply from the collateral vessels of PCA to the anterior cerebral artery and the middle cerebral artery, the lower the possibility of patients suffering from ischemic stroke and intracranial hemorrhage [21, 22]. For this reason, the higher the PSV of PCA, the more collateral circulation would be formed, and a lower incidence of stroke would occur in MMD patients. We also measured the ultrasound parameters of the STA and the MA in MMD patients, but there were no statistically significant differences in these parameters. It can be speculated that the collateral vessels formed by the STA and the MA are not as abundant as the PCA, and their compensatory effects was less pronounced compared with the PCA [23].

There are some limitations in our study. First, we divided MMD patients into a stroke group and a non-stroke group according to clinical manifestations and did not further classify the stroke group into an ischemic stroke group and a hemorrhagic stroke group. Since the occurrence of hemorrhagic stroke in MMD patients is mainly ascribed to the rupture of moyamoya vessels and Willis aneurysms compensating for cerebral ischemia, we did not further divide the stroke group into an ischemic stroke group and a hemorrhagic stroke group. Second, in our study, MMD patients did not receive ultrasound examination before stroke, and future prospective cohort studies are needed to verify the clinical value of our findings.

## Conclusion

Ultrasound parameters are independently correlated with the occurrence of ipsilateral stroke in MMD patients, and ultrasound provides a new approach for identifying the occurrence of stroke in MMD patients. In the future, large-scale prospective cohort studies are needed to verify the clinical value of ultrasound in identifying MMD patients at high risk of stroke.

## Data Availability

The datasets used and/or analyzed during the current study are available from the corresponding author on reasonable request.

## Abbreviations

MMD: Moyamoya disease
ICA: Internal carotid artery
DSA: Digital subtraction angiography
PCA: Posterior cerebral artery
MA: Maxillary artery
STA: Superficial temporal artery
CT: Computed tomography
ID: Internal diameter
PSV: Peak systolic velocity
ICC: Interclass correlation coefficient
